# The influence of liver transplantation on the interplay between gut microbiome and bile acid homeostasis in children with biliary atresia

**DOI:** 10.1097/HC9.0000000000000151

**Published:** 2023-05-15

**Authors:** Birgit Waldner, Denise Aldrian, Thomas Zöggeler, Herbert Oberacher, Rupert Oberhuber, Stefan Schneeberger, Franka Messner, Anna M. Schneider, Benno Kohlmaier, Roland Lanzersdorfer, Wolf-Dietrich Huber, Andreas Entenmann, Thomas Müller, Georg F. Vogel

**Affiliations:** 1 Department of Paediatrics I, Medical University of Innsbruck, Innsbruck, Austria; 2 Institute of Legal Medicine and Core Facility Metabolomics, Medical University of Innsbruck, Innsbruck, Austria; 3 Department of Transplant Surgery, Medical University of Innsbruck, Innsbruck, Austria; 4 Department of Pediatrics, Salzburger Landeskliniken and Paracelsus Medical University, Salzburg, Austria; 5 Department of General Paediatrics, Medical University of Graz, Graz, Austria; 6 Department of Paediatrics and Adolescent Medicine, Johannes Keppler University Linz, Linz, Austria; 7 Department of Pediatrics and Adolescent Medicine, Medical University of Vienna, Vienna, Austria; 8 Institute of Cell Biology, Biocenter, Medical University of Innsbruck, Innsbruck, Austria

## Abstract

Biliary atresia (BA) causes neonatal cholestasis and rapidly progresses into cirrhosis if left untreated. Kasai portoenterostomy may delay cirrhosis. BA remains among the most common indications for liver transplantation (LT) during childhood. Liver function and gut microbiome are interconnected. Disturbed liver function and enterohepatic signaling influence microbial diversity. We, herein, investigate the impact of LT and reestablishment of bile flow on gut microbiome–bile acid homeostasis in children with BA before (pre, n = 10), 3 months (post3m, n = 12), 12 months (post12m, n = 9), and more than 24 months (post24 + m, n = 12) after LT.

**Methods:**

We analyzed the intestinal microbiome of BA patients before and after LT by 16S-rRNA-sequencing and bioinformatics analyses, and serum primary and secondary bile acid levels.

**Results:**

The gut microbiome in BA patients exhibits a markedly reduced alpha diversity in pre (*p* = 0.015) and post3m group (*p* = 0.044), and approximated healthy control groups at later timepoints post12m (*p* = 1.0) and post24 + m (*p* = 0.74). Beta diversity analysis showed overall community structure similarities of pre and post3m (*p* = 0.675), but both differed from the post24 + m (*p* < 0.001). Longitudinal analysis of the composition of the gut microbiome revealed the *Klebsiella* genus to show increased abundance in the post24 + m group compared with an age-matched control (*p* = 0.029). Secondary bile acid production increased 2+ years after LT (*p* = 0.03). Multivariable associations of microbial communities and clinical metadata reveal several significant associations of microbial genera with tacrolimus and mycophenolate mofetil–based immunosuppressive regimens.

**Conclusions:**

In children with BA, the gut microbiome shows strongly reduced diversity before and shortly after LT, and approximates healthy controls at later timepoints. Changes in diversity correlate with altered secondary bile acid synthesis at 2+ years and with the selection of different immunosuppressants.

## INTRODUCTION

Biliary atresia (BA) is a rare neonatal disease and remains among the most common indications for LT during childhood.^1^,^2^ Characterized by inflammation and fibrotic obstruction of the biliary tree, BA causes neonatal cholestasis and rapidly progresses to cirrhosis and end-stage liver disease if left untreated.^1^ Ideally, a hepatoportoenterostomy (Kasai portoenterostomy, KPE) is performed during the first months of life and attains biliary drainage to the intestine. While some patients respond well to this surgical treatment, a marked proportion fails to restore liver function, and thus, liver transplantation (LT) is indicated.^1^

Although the pathophysiology of BA remains to be fully elucidated, selected studies have been recently added to the understanding of the disease.^1^,^3^–^5^

The gut microbiome, with its plethora of enzymatic functions, not only helps to maintain intestinal homeostasis and digestive function but also produces several metabolites that regulate the metabolism of the host.^6^ Bile acids, although primarily produced in the liver, are subsequently metabolized by the gut microbiota into secondary bile acids. Hence, host metabolism can be affected not only by the changes in the composition of the microbiota but also by microbial changes in bile acids that lead to altered signaling through bile acid receptors.^7^,^8^ In BA patients, cholestasis has been shown to be inversely correlating with microbial diversity. KPE resulted in increased *Bifidobacterium* abundance.^9^ Microbial dysbiosis also has prognostic relevance and, interestingly, KPE does not fully alleviate dysbiosis.^10^ Alpha diversity before KPE has been shown to negatively correlate with clearance of jaundice after KPE and differential abundances of *Acinetobacter*, *Clostridiaceae*, *Enterobacteriaceae,* and *Salmonella* have been noticed in BA patients with or without successful clearance of jaundice after KPE.^11^

Liver function and gut microbiome are profoundly interconnected; disturbed liver function and enterohepatic signaling influence microbial diversity.^12^ After LT, dysbiosis persists, but microbial homeostasis continues to adopt.^13^,^14^

As the intestinal microbiome develops over the first months and years of life,^15^,^16^ a disrupted gut-liver axis due to BA, for example, might have severe implications for microbial diversity even after LT. However, pediatric studies are lacking so far.

In this study, we characterize the longitudinal changes in the composition of intestinal microbiome before and after LTs in BA in a pediatric cohort by investigating intrasubject and intersubject microbial diversities by alpha and beta diversity measures. In addition, we study the influence of LT on the interplay between the microbiome and bile acid homeostasis.

## METHODS

### Ethics and patient inclusion

This study was approved by (Nr. 1029/2017) and conducted conforming to the guidelines of the Institutional Review Board of the Medical University of Innsbruck, the 2013 Declaration of Helsinki, and the 2018 Declaration of Istanbul. Written consent was given in writing by all individuals and legal guardians. All patients were referred to the Medical University of Innsbruck for LT as a consequence of BA. After KPE, patients received trimethoprim/sulfamethoxazole or amoxicillin/clavulanic acid. Following LT, all patients received antibiotic therapy for a median of 21 days (interquartile range 6) with piperacillin/tazobactam (82%) or meropenem (18%). Age-matched patients without comorbidities and administration of antibiotics served as controls.

### Patients and sample collection

Fecal samples were collected in sterile plastic tubes and immediately stored at −80 °C until further analysis. Fastened whole blood samples were centrifuged, and the supernatant was collected and immediately stored at −80 °C until further analysis.

### Fecal DNA extraction, sequencing, and sequence analysis

Fecal sample (250 mg) was used to extract DNA with QIAmp PowerFecal Pro DNA kit (Qiagen, Venlo, the Netherlands). DNA was sent for library preparation of the 16S V3-V4 region (forward primer: ACTCCTACGGGAGGCAGCAG and reverse primer: GGACTACHVGGGTWTCTAAT) and sequencing using an Illumina Hiseq. 2500 platform (Illumina, Eindhoven, the Netherlands) at BGI company (Shenzen, China). 16S rRNA raw sequence data were trimmed for barcodes and primers using Cutadapt.^17^

### 16S bioinformatics analysis

DADA2^18^ and R^19^ were used for sequence quality control with filtering of low-quality sequences and chimeras, sequence trimming, and the construction of amplicon sequence variants. Taxonomy was assigned to amplicon sequence variant using the naive Bayesian classifier against Silva operational taxonomic units. Phyloseq^20^ was used for all downstream analysis of 16S taxonomic data, and plots were made with the ggplot2 package. Before analysis, optimal thresholds were determined in phyloseq, and the data were filtered using a prevalence threshold of 4, an abundance threshold of 10, and a max threshold of 5. Only bacteria were kept in the analysis. Further details (eg, alpha diversity, beta diversity, differential abundance, bioinformatic prediction of metabolic capacity, and multivariable analysis) are found in Supplemental Methods, Supplemental Digital Content 1, http://links.lww.com/HC9/A280.

### Alpha/beta diversity

Alpha diversity was calculated based on "Observed", "Chao1", "Shannon" and "Simpson" diversity measures using the phyloseq implementations. The Mann-Whitney U test was applied for testing, and a *p*-value < 0.05 was considered significant. Beta diversity was visualized as nonmetric multidimensional scaling (NMDS) plot on the Bray-Curtis distance that is calculated after performing variance stabilizing transformation. Beta diversity was tested using adonis (permutational multivariate ANOVA using distance matrices) with the R package vegan, and a *p*-value < 0.05 was considered significant. Longitudinal analysis was performed using area plots and the R packages: ggplot2 and microbiomeutilities.

### LEfSe and DESeq. 2

Differential gene analysis was performed using the linear discriminant analysis effect size (LEfSe) and differential expressed genes sequencing (DESeq. 2).^1^,^2^ The pregroup was compared with the post3m, post12m, and post24m groups. In addition, the post24m group was compared with the healthy control group (p24 + m control).

LEfSe input was created using the phyloseq. The 2lefse function from the phyloseq companion package with the order level was chosen for the output. The LEfSe analysis was performed using the online server of the Hutenhower lab.^3^ The timepoint was used as a class, no subclass was used, and per-sample normalization of the sum of the values to 1M was applied. When calculating the LDA effect size, the alpha value for the Kruskal-Wallis test was set to 0.5. LDA scores and the corresponding cladograms were plotted directly on the Hutenhower server.

The DESeq. 2 analysis was performed at the order level as well, and the output was visualized in the volcano plot form.

### PICRUSt2 and ALDEx2

The phylogenetic investigation of communities by reconstruction of unobserved states 2 (PICRUSt2) package was used to infer the enzymatic capacities of microbial communities.^16^ The ANOVA-like differential expression (ALDEx2) compositional data analysis was used to perform differential abundance testing using the Wilcoxon rank test between the pre-post3m, pre-post12m, pre-post24m, and healthy-post24m groups. Raw *p*-values were corrected using the Benjamini-Hochberg correction.^4^

### Multivariable association analysis of metadata

Association analysis of taxonomic abundance on the family level with sample metadata was done using microbiome multivariable association with linear models (MaAsLin2).^5^ Fixed effects were immunosuppression, sex, and urbanicity with reference to the healthy cohort. The difference in taxonomic abundances was deemed significant when q < 0.05.

### Quantification of primary and secondary bile acids

Primary and secondary bile acids in serum samples were quantified with the AbsoluteIDQ Bile Acids kit (Biocrates Life Sciences AG, Innsbruck, Austria). Details of measurements, including the gradient table (Supplementary Table S2), and bioinformatic analysis are found in the Supplemental Methods, Supplemental Digital Content 1, http://links.lww.com/HC9/A280.

### mixOmics—canonical correlation analysis

The phyloseq data were filtered to match the samples, for which bile acid concentration was measured. The canonical correlation analysis between bile acid concentration and microbial families was performed using the shrinkage rCCA method implemented in the mixOmics package.^21^

### Data availability statement

All sequence files are available on the European Nucleotide Archive (ENA) accession PRJEB57590.

## RESULTS

### Study cohort

For this study, we included BA patients who are previously treated with KPE. They were grouped in reference to time: before LT (pre): 10 patients (5 males, 5 females); at a median of 3 months after LT (post3m): 11 patients (6 males, 5 females); at a median of 12 months after LT (post12m): 9 patients (5 males, 4 females); and at a median of 59 months after LT (post24 + m) 12 (6 males, 6 females). An age-matched group (control) of 19 healthy children (10 males, 9 females) was used for comparison (general metrics in Table 1, age-matched groups in Supplemental Methods, Supplemental Table S1, Supplemental Digital Content 1, http://links.lww.com/HC9/A280). For longitudinal analysis, 8 patients were followed over 3 timepoints, and 3 of the 8 were followed over an additional fourth timepoint.

**TABLE 1:** Composition and characteristics of the study cohort

	Pre (n = 10)	Post3m (n = 11)	Post12m (n = 9)	Post24 + m (n = 12)	Control (n = 19)
Female, n (%)	5 (50)	5 (45)	4 (45)	6 (50)	9 (47)
Median age in months (IQR)	7 (2.25)	10 (3)	19 (4)	65 (60.25)	72 (90)
Diet					
Heparon, n (%)	4 (40)	6 (55)	—	—	—
Breastmilk, n (%)	6 (60)	5 (45)	—	—	—
Breastmilk only n (%)	4 (40)	2 (18)	—	—	—
Solid food, n (%)	3 (30)	7 (64)	9 (100)	12 (100)	19 (100)
Medium-chain triglycerides, n (%)	3 (27)	—	—	—	—
Urbanicity					
Rural, n (%)	5 (50)	6 (55)	5 (55)	5 (42)	11 (58)
Urban, n (%)	5 (50)	5 (45)	4 (45)	7 (58)	8 (42)
Delivery method					
Vaginal, n (%)	8 (80)	10 (91)	7 (78)	8 (67)	—
C-section, n (%)	2 (20)	1 (9)	2 (22)	4 (33)	—
Median age at KPE (IQR)	1.5 (1)	1 (1)	2 (1)	1 (1)	—
Median age at LT in months (IQR)	7.5 (2.5)	7 (3)	7 (3)	8 (1.75)	—
Median months after LT (IQR)	—	3 (1)	12 (2)	59 (57)	—
Median distance KPE to LT in months (IQR)	—	5 (3)	4 (3)	7 (1)	—
LD, Split Graft (%)	-	11 (100)	9 (100)	9 (75)	-
DD, full size graft (%)	—	0	0	3 (25)	—
Total bilirubin, µmol/L (IQR)	187.42 (125.6)	4.615 (2.95)	5.99 (3.24)	8.72 (4.44)	—
AST, U/L (IQR)	167.5 (40.5)	35 (11.5)	44 (8)	44.5 (11.25)	—
ALT, U/L (IQR)	85 (23.5)	37 (16)	32 (8)	26 (13.5)	—
GGT, U/L (IQR)	260 (189.75)	58 (33.5)	19 (12)	19.5 (30.5)	—
alk. Phos (IQR)	842 (163)	354 (227.5)	309 (115)	243 (192.75)	—
bile acids (IQR)	304.8 (198.65)	5.9 (8.05)	13.5 (18.8)	21 (33.25)	—
Albumin, g/L (IQR)	30.775 (8.45)	42.48 (2.28)	42.9 (4.85)	41.15 (6.14)	—
Platelet count, 10^9^/L (IQR)	193.5 (96.25)	276 (77)	264 (127)	200.5 (100.5)	—
Cholangitis, n (%)	0	1 (9)	1 (11)	1 (8)	0
Antibiotics, n (%)	6 (60)	11 (100)	1 (11)	1 (8)	0
Median length of antibiotic treatment in days (IQR)	—	21 (6)	—	—	—
UDCA, n (%)	10 (100)	5 (45)	3 (33)	1 (8)	0
Add-on MMF, n (%)	—	1 (9)	1 (11)	2 (17)	—
Complications after LT, n (%)	—	4 (36)	5 (56)	9 (75)	—
Vascular, n (%)	—	0	0	6 (50)	—
Biliary, n (%)	—	1 (9)	1 (11)	2 (17)	—
Rejection, n (%)	—	2 (18)	2 (22)	3 (25)	—
Infectious, n (%)	—	1 (9)	2 (22)	1 (8)	—

Abbreviations: ALT indicates alanine aminotransferase, AST, alkaline phosphatase, GGT, gamma glutamyl transpeptidase, IQR, interquartile range; KPE, Kasai portoenterostomy, LT, liver transplantation, MMF, mycophenolate mofetil, UDCA, ursodeoxycholic acid. Values are given as median and respective interquartile range (IQR) if not stated otherwise.

Patients in the pregroup were dominantly breast-fed (60%), 4 out of 6 were fully breast-fed, and 2 were received complementary feeding. In the post3m group, the proportion of breast-fed children is still 45%. All other patients and controls received a formula or a regular solid food-based diet. Urbanicity and mode of delivery were comparable between different patient groups. The antibiotic treatment was given in BA patients pre (60%), post3m (100%), post12m (11%), and post24 + m (8%) groups. The ursodeoxycholic acid (UDCA) treatment was reported in BA patient pre (100%), post3m (45%), post12m (33%), and post24 + m (8%) groups. After LT, the immunosuppressive regimen was tacrolimus-based. Tacrolimus with mycophenolate mofetil (MMF) was used in 1 (9%) patient in the post3m, 1 (11%) patient in the post12m, and 2 (17%) patients in the post24 + m group. Post-LT complications were seen in post3m (36%), post12m (56%), and post24 + m (75%). In the post3m group, rejection was the main complication (18%), followed by biliary (9%) and infectious (9%) complications. No vascular problems were seen in this group. In the post12m group, infections (22%) and rejection (22%) were the leading causes of complications, followed by biliary leakage (11%); again, no vascular problem was seen. On the other hand, in the post24 + m group, vascular problems (50%), more precise portal vein obstructions, were noted. Also, rejection (25%), biliary leakage (17%), and infectious complications (8%) (eg, recurrent cholangitis) were observed in this group. See Table 1 for characteristics, laboratory tests, and post-transplant course of patients and groups.

### Gut microbiome alpha and beta diversity analysis

**FIGURE 1:**
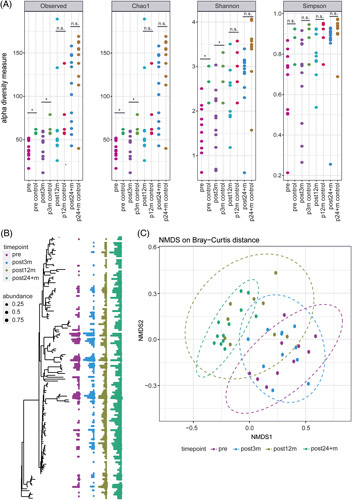
Alpha and beta diversities, and abundance analysis of studied groups. (A) Alpha diversity of gut microbiota differed in pre and post3m groups, but approximated healthy control groups at later timepoints post12m and post24 + m. Different alpha diversity indices are shown. (B) Phylogenetic abundance and richness on the genus level are compared across all groups (colors). Taxonomic abundance was reduced in pre and post3m groups and increased over time in post12m and post24 + m groups. (C) Beta diversity is shown with NMDS using Bray-Curtis distance measures. Pre and post3m cluster together and differ markedly from post24 + m samples. Abbreviations: NMDS, nonmetric multidimensional scaling.

To overcome the age bias of a developing gut microbiome, we compared all groups to age-matched control. Alpha diversity of gut microbiota differed in pre (*p* = 0.015) and post3m groups (*p* = 0.044) but approximated healthy control groups at later timepoints in post12m (*p* = 1.0) and post24 + m (*p* = 0.74) (Figure 1A). Pre and post3m groups showed decreased alpha diversity compared with post24 + m (*p* = 0.001 and 0.01) (Figure 1A).

Taxonomic abundance was reduced in pre and post3m groups and increased over time in post12m and post24 + m groups (Figure 1B). The beta diversity analysis by Bray-Curtis distance metrics showed overall community structure similarities of pre and post3m (*p* = 0.6753), but the community structure differed between post3m and post12m (*p* = 0.0175), and post12m and post24 + m (*p* = 0.01334) (Figure 1C). With respect to age-matched controls, the overall community structure was similar in the pre (*p* = 0.1942), post3m (*p* = 0.1149), and post12m (*p* = 0.7163) groups but strongly differed in the post24 + m group (*p* = 0.0009).

### Longitudinal changes in composition of gut microbiota

**FIGURE 2:**
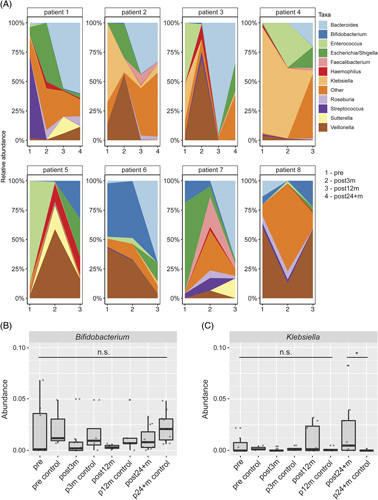
Longitudinal changes in the composition of gut microbiota. (A) Changes in microbial composition in individual patients over time are shown in relative abundances on the genus level. (B) Abundance of the *Bifidobacterium* genus is shown across all groups. (C) Abundance of the *Klebsiella* genus is shown across all groups. (B, C) * = *p* < 0.05, and n.s. = not significant.

To gain better insight into individual changes in gut microbiome composition, taxonomic changes in the genus level were analyzed longitudinally for 8 individual patients for at least 3 timepoints (pre to post12m) (Figure 2A). Here, a common pattern was the increase in *Bacteroides* over time in the majority of patients (Figure 2A). The beta diversity analysis by Bray-Curtis distance metrics on the longitudinally tracked subcohort showed overall community structure similarities of pre and post3m (*p* = 0.5371), but the community structure differed between post3m and post12m (*p* = 0.0297), and post12m and post24 + m (*p* = 0.0458). This pattern was comparable to the differences in the community structure of the entire groups mentioned before.

To overcome individual bias in the taxonomic analysis, we further investigated the abundances of selected genera in greater detail. No differences in abundances of *Bifidobacterium* were seen at all timepoints and correlated with healthy controls (*p* = 1), whereas *Klebsiella* genus showed increased abundance in the post24 + m compared with age-matched healthy controls (*p* = 0.029) (Figure 2C). In addition, abundances of other genera associated with end-stage liver disease, for example, *Clostridium* and *Enterococcus*, or putatively beneficial genera, such as *Roseburia*, did not show significant differences in comparison to age-matched controls (Supplemental Figure S1A–C).

### Differential abundance and LEfSe of gut microbiota

**FIGURE 3:**
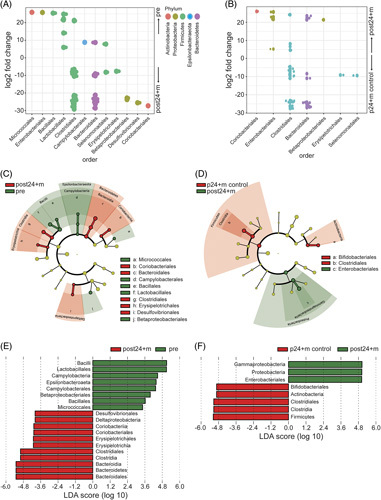
Differential abundances and linear discriminant effect size analysis (LEfSe) of gut microbiota. (A) Dotplot of the DESeq. 2 analysis for differential taxa abundances of pre and post24 + m groups on taxonomic order level. Phylum classifications are annotated in different colors. (B) Dotplot of the DESeq. 2 analysis for differential taxa abundances of post24 + m and age-matched healthy control groups (p24 + m control) on the taxonomic order level. Color annotation of phyla is the same as in (A). (C) Cladogram of LEfSe analysis results of pre and post24 + m groups on the taxonomic order level. Over-representation of taxa is color-coded in red (post24 + m) and green (pre). Radiating circle depicts phylum to order level. (D) Cladogram of LEfSe analysis results of post24 + m and age-matched control groups (p24 + m control) on the taxonomic order level. Over-representation of taxa is color-coded in red (p24 + m control) and green (post24 + m). Radiating circle depicts phylum to order level. (E) Bar chart of LDA values with significantly different abundances to order level is shown comparing pre versus post24 + m, and healthy versus post24 + m in (F). Abbreviation: LDA, linear discriminant analysis.

As gut microbial intracommunity and intercommunity abundances differed between patient groups and controls, we further examined which taxa showed the strongest differences. Therefore, we applied 2 independent tools, DESeq. 2 and discriminant effect size analysis (LEfSe), to test differential abundance on taxonomic order level between pre and post24 + m, as well as between post24 + m and age-matched controls. The DESeq. 2 analysis found *Bacillales* and *Lactobacillales* to be enriched in pregroup patients, whereas *Clostridiales* and *Bacteroidales* concentrated in post24 + m communities (Figure 3A). This finding was confirmed to be independent of the LEfSe analysis (Figure 3C and E). This pattern changed when comparing post24 + m with controls. Here, the DESeq. 2 analysis showed that *Clostridiales* and *Bacteroidales* are now enriched in the control group (Figure 3B). The LEfSe analysis attributed *Clostridiales* and *Bifidobacteriales* to the control group and *Enterobacteroidales* to the post24 + m group (Figure 3D and F).

### Prediction of metabolic pathways

The gut microbiome fulfills various metabolic functions. To assess the metabolic capacity of the characterized communities, we used the PICRUSt2 package. The resulting metagenomics prediction was further subjected to the ALDEx2 analysis, which allowed us to test for the differential abundance of metabolic pathways in pre and post24 + m, and post24 + m and age-matched controls.

**FIGURE 4:**
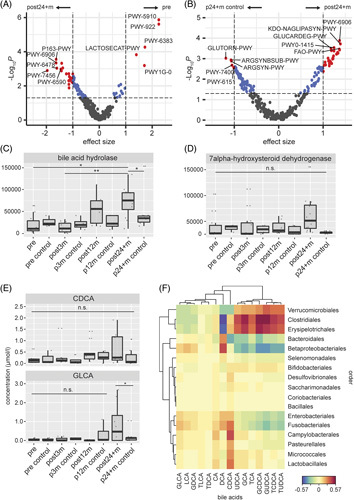
Differential abundance of microbiota-associated metabolic pathways. (A) Volcano plot of the ALDEx2 analysis for differential abundances of metabolic capacity of gut microbiota in pre and post24 + m groups. The top 5 upregulated and downregulated MetaCyc pathways are annotated. (B) Volcano plot of the ALDEx2 analysis for differential abundances of metabolic capacity of gut microbiota in post24 + m and age-matched (p24 + m control) groups. The top 5 upregulated and downregulated MetaCyc pathways are annotated. (A, B) Vertical dashed lines represent the effect size cutoff, and horizontal dashed lines represent the *p* < 0.05 cutoff. Pathways above the effect size cutoff are represented by blue dots and above both effect size and *p* < 0.05 cutoff by red dots. (C) Comparison of the phylogenetic investigation of communities by reconstruction of unobserved states (PICRUSt2) analysis of KEGG metabolic pathway abundance of bile acid hydrolase and (D) 7alpha-hydroxysteroid dehydrogenase. (E) Serum CDCA, GDCA, and GLCA in µmol/L. (C, D, E) * = *p* < 0.05, ** = *p* < 0.01, and n.s. = not significant. (F) Canonical correlation analysis for 15 selected bile acids and microbial order abundances. Positive correlations are on the red spectrum, while negative correlations are on the blue spectrum. Abbreviations: CDCA, concentration of chenodeoxycholic acid; GDCA, glycodeoxycholic acid; GLCA, glycolithocholic acid.

The difference in community composition of pre and post24 + m resulted in several differentially represented metabolic pathways, which is associated with bacterial metabolic processes (Figure 4A, Supplemental Figure 2A). When comparing post24 + m and age-matched controls, we found significantly enriched pathways in the post24 + m group and the age-matched controls (Figure 4B, Supplemental Figure S2B).

We further studied metabolic pathways with relevance to the gut-liver axis. Therefore, we selectively compared abundances of Kyoto Encyclopedia of Genes and Genomes (KEGG) pathway terminology for secondary bile acid synthesis. Bile acid hydrolase was found to be increased in the post24 + m group in comparison to age-matched controls, and pre and post3m groups (*p* = 0.045, 0.011, and 0.002, respectively) (Figure 4C), whereas no significant differences were seen in 7alpha-hydroxysteroid dehydrogenase (Figure 4D).

### Bile acid metabolomics

Targeted metabolomic analysis of serum bile acids showed chenodeoxycholic acid (CDCA) to be elevated in the post24 + m group (Fig. 4E) with no significant difference in comparison to other timepoints and controls. In contrast, for glycolithocholic acid (GLCA), there was a significant difference between the post24 + m group and age-matched controls (*p* = 0.03). Furthermore, a pronounced cholestatic phenotype in the pregroup was found (Supplemental Figure S2B). The NMDS plot on the Bray-Curtis distance of the bile acids (Supplemental Figure S2C) revealed a similar picture as the NMDS plot of the microbiota (Figure 1C), indicating a relation between the microbiome and bile acid metabolism.

The canonical correlation analysis was performed for the investigation of the correlation between 15 selected bile acids and the microbial families, which is associated with secondary bile acid synthesis. Glycocholic acid (GCA), glycochenodeoxycholic acid (GCDCA), and glycoursodeoxycholic acid (GUDCA) seem to be highly correlated to *Clostridiales.* In addition, GCA and GCDCA correlated with *Erysipelotrichales*, whereas deoxycholic acid (DCA) showed negative correlations with *Clostridiales and Erysipelotrichales*.

*Verrucomicrobiales*, *Clostridiales*, and *Erysipelotrichales* showed correlations with bile acids in opposite directions compared with *Bacteriodales*, *Betaproteobacteriales*, *Enterobacteriales*, and *Fusobacteriales*. Less strong correlations to individual bile acids are found for *Selenomonadales*, *Bifidobacteriales*, *Desulvovibrionales*, *Saccharimonadales*, *Coriobacteriales*, *and Bacillales* (Figure 4F).

### Association of gut microbial abundances with clinical factors

**FIGURE 5:**
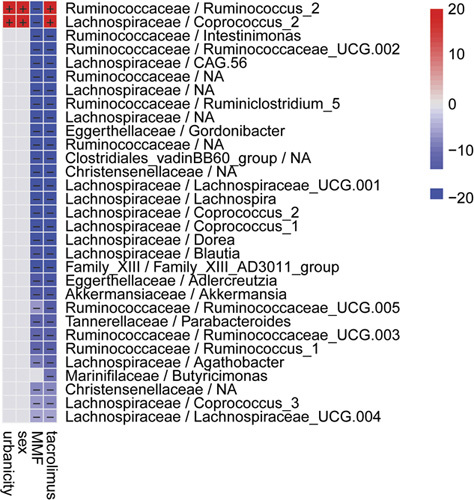
Association of gut microbiota with sample metadata. (A) Heatmap summarizing MaAsLin2 multivariable analysis gut microbial abundance and sample metadata (eg, urbanicity, sex, immunosuppression with tacrolimus and mycophenolate mofetil (MMF), and tacrolimus alone). Positive associations are shown in red and negative ones in blue. Associations for urbanicity positively correlate with the factor urban and for sex with the factor male. Microbiota is shown in family/genus. Color key: −log (qvalue) * sign (coefficient).

As the individual microbiome can be influenced by environmental factors, we applied MaAsLin2 to test multivariable associations of the observed microbial communities and clinical metadata. Significant associations with the immunosuppressive regimen were found. *Ruminococcus_2* and *Coprocooccus_2* were positively associated with tacrolimus alone but negatively in patients with added MMF (n = 4) (Table 1). Also, *Butyricimonas* was only associated with tacrolimus but not with tacrolimus and MMF (Figure 5A). Both genera, *Ruminococcus_2* and *Coprocooccus_2*, were also positively associated with the factor urbanicity/urban and sex/male (Figure 5A). Of note, the type of liver graft (eg, full size or split graft) or breastfeeding did not yield significant associations in this cohort.

## DISCUSSION

Studying the composition of the gut microbiota with its complex interaction with bile acid homeostasis and correlating it with clinical cofactors is a challenging endeavor. BA with failed response to KPE poses an auspicious model to study the interaction between bile acid metabolism and gut microbiome, as bile flow is markedly reduced before and abruptly restored right after LT. Here, we characterized the longitudinal compositional changes in the intestinal microbiome before and after LTs in pediatric individuals with BA in comparison to age-matched healthy controls and the influence of LT on the interplay between the microbiome and bile acid homeostasis. We demonstrated that LT partially restored the composition of the intestinal microbiome with increasing microbial diversity, but differences in microbial composition were still present 2+ years after transplant compared with healthy controls. Furthermore, the metabolic capacity of microbiota identified was investigated and targeted bile acid metabolomics were performed. We were able to show that secondary bile production of microbiota was found highest in the post24 + m group. Multivariable associations of the observed microbial communities and specific clinical metadata factors predominantly revealed significant associations with immunosuppressive regimens.

This study is the first to examine the impact of LT on the gut-liver axis in a pediatric cohort, highlights microbial compositional data and associations with LT-linked clinical factors that are influencing the microbiome, and describes the associations between bacterial commensals and bile acid metabolism. However, there are some limitations to the research: the small sample size, taxonomic depth limited by 16S-rDNA-sequencing, the fact that we can only provide single-center data, and the associative nature of results certainly limit the power of the study. Despite these limitations, significant changes in the gut-liver axis were observed.

Usually characterized by decreased diversity, an increase in potentially pathogenic, and a decrease in beneficial taxa compared with healthy microbiota, microbial dysbiosis indicates an imbalance in the gut microbial community and can be associated with various liver diseases.^22^–^26^ The altered microbial composition has also been observed in pediatric patients with BA.^10^,^27^ These results have been confirmed by our data; the gut microbiome in individuals with BA with failed response to KPE exhibited a markedly reduced alpha diversity before and 3 months after LT compared with their age-matched healthy controls. Alpha diversity, especially in fully breast-fed infants, can already be reduced for physiological reasons. Compared with formula-fed infants, breast-fed infants harbor a fecal microbiota that is more than 2 times increased in numbers of *Bifidobacteria*.^28^ The ratio of breast-fed to formula-fed infants is comparable in the pre and post3m groups, but comparison with the healthy control group is limited because of the average age of 12 months and the different dietary forms (solid food). Interestingly, in a longitudinal analysis, no differences in abundances of the *Bifidobacterium* genus were seen in pre and post3m groups in comparison with correlated controls. This means that, even though about half of the patients in the pre and post3m groups were at least additionally breast-fed, there was no difference in the abundance of *Bifidobacteria* compared with the control group fed with solid food. Recent studies showed that *Bifidobacteria* correlate negatively with cholestasis and may serve as a predictor for successful KPE.^27^,^29^,^30^

The beta diversity analysis showed overall community structure similarities of pre and post3m, but both differed from the post24 + m. To gain better insight into individual changes in gut microbiome composition over time, we further investigated the abundances of selected genera, which are associated with liver disease in greater detail. No differences in comparison to age-matched healthy controls were seen in *Clostridium* or *Enterococcus* genera, whereas *Klebsiella* genera showed increased abundance in the post24 + m compared with age-matched healthy controls. An increase in *Klebsiella* abundance is linked with liver disease.^31^ However, the clinical relevance of this finding remains uncertain.

We have also investigated major differences in microbial composition between groups in an unbiased fashion by differential abundance analysis using 2 independent methods: DESeq. 2 and LEfSe. Most of our results were consistent with both methods. However, when comparing post24 + m with controls, the DESeq. 2 analysis showed that *Clostridiales* and *Bacteroidales* are enriched in the control group. The LEfSe analysis attributed *Clostridiales* and *Bifidobacteriales* to the control group and *Enterobacteroidales* to the post24 + m group. This is expected when using differential abundance methodologies, which is why our biological interpretation focused on the consensus of DESeq. 2 and LEfSe results.^32^

The surgery itself temporarily increases intestinal permeability resulting from a disrupted intestinal barrier and allows pathogenic bacteria to enter the portal circulation.^33^ Conversely, the administration of antibiotics and immunosuppressants in the perioperative period leads to a decrease in the diversity of the intestinal microbiota.^34^,^35^ Accordingly, an impairment in the compositional balance of the microbiome in the early post-transplant setting is to be expected. We showed that alpha and beta diversities are significantly altered before but also short after LT compared with 1 year after. Despite the normalization of alpha diversity, the composition of gut microbiota of the post24 + m group still remains different from the healthy control group. In conclusion, restoring normal liver function may partially improve altered microbial composition and function.

A balanced gut microbiome is essential for maintaining the normal liver function. Therefore, it is even more important to identify the influencing factors that contribute to dysbiosis to be able to modify them if necessary. We applied MaAsLin2 to test multivariable associations of the observed microbial communities and specific clinical metadata factors, and were able to illuminate significant associations with different immunosuppressive regimens. The alteration of the gut microbiome by immunosuppressive agents used in solid organ transplantation has been investigated before. In a systematic review, 70% of the articles indicated changes in quantities of anaerobic bacteria, including *Ruminococcaceae*, *Lachnospiraceae*, *Firmicutes*, *Bacteroides*, and *Clostridiales*.^36^ Corresponding to these results, we found the genera *Ruminococcus_2* and *Coprocooccus_2* to be positively associated with tacrolimus alone but negatively in patients with added MMF. Also, *Butyricimonas* was only associated with tacrolimus but not with tacrolimus and MMF. It has been shown before that tacrolimus-induced dysbiosis results in functional alterations of the gut microbiota, and these alterations can also result in aggravating some of the drugs’ side effects.^37^ The impact of immunosuppressive therapy on the observed changes needs to be further studied, especially in a pediatric cohort. As the individual microbiome can be influenced by other environmental factors, we also had a look at the type of liver graft (eg, full size or split graft) or breastfeeding, which both did not yield significant associations in our cohort.

Besides the external influences, the bile acid pool is an essential modulator of the gut microbiome and vice versa.^16^,^38^ Through the conjugation of secondary bile acids by the microbiota itself, microbial changes in bile acids can lead to altered signaling through bile acid receptors.^7^,^8^ Conversely, bile acids emerge to be the major regulator to shape the gut microbiome itself. Recent studies indicated that low bile acid levels entering the intestine are linked to dysbiosis in progressive cirrhosis.^39^

In our metagenomic analysis, the difference in community composition before and after LTs yielded several differentially represented metabolic pathways, which are associated with bacterial metabolic processes. Bile acid hydrolase, a key enzyme for secondary bile production of microbiota, was found to increase in post24 + m groups in comparison to pre and post3m groups. In line, we found the secondary conjugated bile acid GLCA, synthesized in the gut, actively reabsorbed in the terminal ileum,^8^ and increased in serum samples of the post24 + m group. The canonical correlation analysis between different bile acid concentrations and microbial taxa revealed that *Verrucomicrobiales, Clostridiales,* and *Erysipelotrichales* correlate with various bile acid species. Many of them harbor species capable of modifying bile acids.^8^,^15^,^16^,^38^,^40^ However, we cannot conclude whether restored bile acid secretion or altered microbiome diversity results in the observed changes. The measured increase in GLCA serum levels could have pleiotropic effects on the hosts’ bile acid metabolism itself, energy metabolism, apoptosis, and oxidative stress and is excellently reviewed.^38^ Yet, further studies with larger cohorts are required to elucidate this delicate interplay.

In conclusion, the gut microbiome showed strongly reduced diversity before and shortly after LT in children with BA. Differences in microbial composition were present 2+ years after transplant compared with healthy controls. Changes in diversity correlated with altered secondary bile acid synthesis at 2+ years and with the selection of different immunosuppressive regimens.

## Supplementary Material
