# Supplementary material for: The influence of liver transplantation on the interplay between gut microbiome and bile acid homeostasis in children with biliary atresia
Source: Hepatol Commun. 2023 May 15;7(6):e0151. doi: 10.1097/HC9.0000000000000151 (PMC10187839; doi:10.1097/HC9.0000000000000151)
Supplement: Supplementary file 1 [file hc9-7-e0151-s001.docx]

**The influence of liver transplantation on the interplay between gut microbiome and bile acid homeostasis in children with biliary atresia**

**Supplementary Methods**

**Patient cohort age and age-matched control groups**

|  | median age (IQR) |
| --- | --- |
| pre | 7 (2.25) |
| pre controls | 12 (0) |
| post3m | 10 (3) |
| p3m controls | 12 (3) |
| post12 | 19 (4) |
| p12m controls | 12 (12) |
| post24+m | 65 (60.25) |
| p24+m controls | 78 (42) |

Supplementary Table 1. Median age of patients and age-matched healthy controls in months. IQR = interquartile range.

**Quantification of primary and secondary bile acids**

Primary and secondary bile acids in serum samples were quantified with the AbsoluteIDQ Bile Acids kit (Biocrates Life Sciences AG, Innsbruck, Austria). To ensure accuracy and precision, the method provides seven calibration standards, a mixture of nine isotope-labeled internal standards, and three quality-control samples.

Analysis was performed as described in manufacturer´s instructions. In short, 10 µL of internal standards mixture were pipetted onto the filter spots suspended in the wells of the 96-well filter plate. This filter plate was fixed on top of a deep-well plate serving as a receiving plate for the extract later on. After drying under nitrogen stream, 10 µL samples were pipetted on the filter spots, followed again by the nitrogen drying. 100 µL methanol was then added to the wells and the plate was shaken for 20 min. The plate was centrifuged to elute the methanol extract into the lower receiving deep-well plate. Finally, the extracts were mixed with 60 µL water and analysed with LC-MS/MS.

The LC-MS/MS system consisted of a Waters ACQUITY UPLC H-Class PLUS Bio (Waters, Manchester, UK) and a QTrap 6500+ mass spectrometer (Sciex, Framingham, MA, USA). Chromatographic separations were accomplished on a reversed-phase column part of the kit. Mobile phase A represented a 10 mM solution of ammonium acetate in water containing 0.015% formic acid. Mobile phase B was a 10 mM solution of ammonium acetate in water containing 30% methanol, 65% acetonitrile and 0.015% formic acid. The gradient table is shown in Supplementary Table 1. The column temperature was held at 50 °C. The injection volume was 10 µL. Mass spectrometry detection was performed with electrospray ionization in negative ion mode. 49 precursor-to-product ion transitions were monitored in scheduled multiple reaction monitoring mode.

Bile acid concentrations were calculated in MetIDQ (Biocrates Life Sciences). All calibration curves had R^2^ values between 0.9982 and 0.9998. The accuracies of standards and quality controls were within 80-120%.

| Time [min] | Flow rate [ml/min] | Mobile phase A [%] | Mobile phase B [%] |
| --- | --- | --- | --- |
| 0.00 | 0.5 | 65 | 35 |
| 0.25 | 0.5 | 65 | 35 |
| 0.35 | 0.5 | 60 | 40 |
| 1.90 | 0.8 | 55 | 45 |
| 2.10 | 0.8 | 45 | 55 |
| 3.30 | 1.0 | 25 | 75 |
| 3.50 | 1.0 | 0 | 100 |
| 4.00 | 1.0 | 0 | 100 |
| 4.10 | 0.9 | 65 | 35 |
| 5.00 | 0.5 | 65 | 35 |

Supplementary Table 2. Gradient table

Values below the limit of quantification were set to 0 for subsequent analysis. An NMDS plot on the Bray-Curtis distance was created for comparison with the beta-diversity plot of the 16S data. Mouse bile acids were excluded from subsequent analyses.
